# A Single-Nucleotide Polymorphism in *ABCC4* Is Associated with Tenofovir-Related Beta2-Microglobulinuria in Thai Patients with HIV-1 Infection

**DOI:** 10.1371/journal.pone.0147724

**Published:** 2016-01-25

**Authors:** Sirirat Likanonsakul, Bussakorn Suntisuklappon, Ravee Nitiyanontakij, Wisit Prasithsirikul, Emi E. Nakayama, Tatsuo Shioda, Chariya Sangsajja

**Affiliations:** 1 Bamrasnaradura Infectious Diseases Institute, Department of Disease Control, Ministry of Public Health, Nonthaburi, Thailand; 2 Department of Viral Infections, Research Institute for Microbial Diseases, Osaka University, Suita, Osaka, Japan; Centro de Biología Molecular Severo Ochoa (CSIC-UAM), SPAIN

## Abstract

**Background:**

In Thailand, the combined generic anti-retroviral drug stavudine/lamivudine/nevirapine (d4T/3TC/NVP) has been used to treat human immunodeficiency virus (HIV)-infected individuals since 2001. Due to relatively frequent adverse effects, d4T gradually has been replaced with tenofovir disoproxil fumarate (TDF). Although the frequency of adverse drug effects with TDF is lower than that with d4T, TDF is known to induce kidney dysfunction, especially in the proximal tubules. It has been reported that renal tubular transporters, including members of the multi-drug resistant (MDR) protein family, are implicated in tenofovir extrusion and may, therefore, confer susceptibility to TDF-induced kidney tubular dysfunction (KTD). We have explored the association between KTD and polymorphisms in genes that encode adenosine triphosphate-binding cassette (ABC)-type MDRs.

**Methods:**

HIV-infected patients receiving TDF-containing antiretroviral regimens for at least one year were enrolled in the study. The levels of beta2-microglobulin in urine and creatinine (Cr) were measured. Three single-nucleotide polymorphisms, *ABCC2* C-24T (rs717620), *ABCC2 G1429A* (rs2273697), and *ABCC4* T4976C (rs1059751), were analyzed using TaqMan SNP genotyping assays.

**Results:**

A total of 273 HIV-infected patients were recruited. The median number of years of TDF treatment was 5.04 with interquartile range (IQR) of 3.9–6.7. Despite the length of treatment with TDF, 98.5% patients maintained an estimated glomerular filtration rate (eGFR) of >60 mL/min as calculated by the CKD-EPI formula. Fifty-four patients (19.8%) showed beta2-microglobulinuria (median 2636 μg/g Cr with IQR of 1519–13197 μg/g Cr). The allele frequency of *ABCC4* T4976C among those 54 patients was 0.602, compared to 0.475 among the 219 remaining patients (p = 0.018).

**Conclusions:**

Approximately 20% of HIV-infected patients receiving TDF showed beta2-microglobulinuria. The C allele at position 4976 of the *ABCC4* gene was associated with beta2-microglobulinuria in this population. This polymorphism may help to identify patients at greater risk for developing TDF-associated KTD.

## Introduction

Kidney tubular dysfunction (KTD) is a recognized complication of the antiretroviral drug nucleotide reverse transcriptase inhibitor tenofovir disoproxil fumarate (TDF) [1–9]. The true incidence rate and onset time of KTD are unknown, since the incidence of KTD ranges from 1.4% [7] to 22% [8]. While preliminary data support the quantification of urinary low molecular-weight proteins such as alpha1- or beta2-microglobulin (B2MG), cystatin C, or retinol-binding protein in assessing KTD [10, 11], it remains unclear whether such a strategy allows the early detection of treatment-limiting KTD.

The mechanism by which tenofovir may cause renal damage is not well understood, although interference with transporter proteins in the renal tubule may play a role. Organic anion transporters (OATs) OAT1 and OAT3 mediate tenofovir uptake into the epithelial cells of the kidney tubule through the basolateral membrane [12, 13]. These transporters are encoded by the *SLC22A6* and *SLC22A8* genes, respectively [14]. Known substrates for OAT1 include cyclic adenosine monophosphate, cyclic guanosine monophosphate, antiviral agents (acyclovir, cidofovir, and zidovudine), antibiotics, and diuretics [15]. OAT1 and OAT3 are expressed in the basolateral membrane, whereas OAT4 (encoded by *SLC22A11*) is expressed in the luminal membrane. Once tenofovir enters tubular cells, secretion of the compound depends on efflux by transporters on the luminal membrane. Although tenofovir uptake from blood into the proximal tubule has been characterized, efflux transport through the luminal membrane is not well studied. Proteins supposed to be involved in tenofovir efflux at the luminal membrane include multi-drug resistance protein 2 (MRP2) and MRP4. These proteins are encoded by the adenosine triphosphate-binding cassette (ABC) genes *ABCC2* and *ABCC4*, respectively. Both MRP2 and MRP4 are energy-dependent pumps that efflux their substrates into the glomerular filtrate [16]. In addition, MRP7 (encoded by *ABCC10* gene) has been shown to transport tenofovir, although the orientation of this transporter in proximal tubule cells has not yet been defined [17, 18]. For many antiviral drugs, efflux at the luminal membrane is rate limiting, in some cases leading to intracellular accumulation of compounds. Therefore, drugs such as cidofovir and adefovir may cause concentration-dependent renal toxicity [19]. Because cidofovir, adefovir, and tenofovir are all nucleotide analogues, tenofovir accumulation within tubular epithelial cells also may interfere with renal function. Moreover, transporter expression may modulate the extent of tubular damage. Variants of *ABCC4* [20, 21], *ABCC2* [20, 22], and *ABCC10* [17] have been shown to be associated with KTD, but polymorphisms in *SLC22A6* and *ABCB1* have not [21, 22]. In addition, old age and lower body weight also have been shown to be risk factors [21–23]. However, the optimal monitoring strategy for KTD in patients receiving TDF has not yet been established.

In Thailand, a combined generic antiretroviral drug of stavudine/lamivudine/nevirapine (d4T/3TC/NVP), GPOvir, has been used to treat human immunodeficiency virus (HIV)-infected individuals since 2001. Due to relatively frequent adverse effects, the Thailand national guideline for HIV treatment was revised in 2010, and d4T gradually has been replaced with TDF. In the present study, we explored the association between levels of urinary B2MG and polymorphisms in *ABC*-type genes encoding MDRs in Thai HIV patients who have been receiving TDF for more than 1 year.

## Materials and Methods

### Patients

This study recruited HIV-1-infected individuals, aged 18 to 60 years, who had been receiving anti-retroviral therapy (ART) including TDF for at least one year at the Bamrasnaradura Infectious Diseases Institute, Nonthaburi, Thailand. Each participant signed an informed consent form. The following patients were excluded from the study: patients with incomplete history of ARV treatment; who were pregnant or lactating; who presented with mental disorder, diabetes, hypertension, heart disease, or kidney disease before receiving TDF; or those who were receiving any other medication known to affect kidney function. Aliquots of whole blood were collected from each patient and stored at -20°C until DNA extraction. The study was approved by the institutional ethical committees at the Bamrasnaradura Infectious Diseases Institute and at the Department of Disease Control, Ministry of Public Health, Thailand.

### Clinical test measurements

Both blood and spot urine samples were collected on the day of enrollment along with body weight measurement. The blood samples were used to measure serum creatinine (sCr) and serum phosphate levels, whereas the urine samples were used to measure protein, phosphate, creatinine, and B2MG levels. B2MG was measured by enzyme-linked fluorescent assay (ELFA) using a Vidas analyzer (bioMérieux, France). Other parameters were measured by photometric assays using c501 Hitachi and Cobas Integra 400 plus analyzers (Roche Diagnostics, Switzerland). The values of B2MG in the urine samples were expressed relative to urinary creatinine of 1 g/L (/g Cr). Estimated glomerular filtration rate (eGFR) was calculated according to the CKD-EPI formula [24], depending on serum creatinine, sex, and age. The fractional excretion of phosphate (FeP; (urine phosphate * serum creatinine) / (serum phosphate * urine creatinine) * 100) and tubular maximal transport of phosphate/estimated glomerular filtration rate (Tmp/eGFR; serum phosphate—(urine phosphate * serum creatinine) / urine creatinine) were calculated from the values of creatinine and phosphate values in serum and urine according to the equations in the above parentheses. CD4 counts were measured by flow cytometry (BD Tritest BD Biosciences, San Jose CA, USA).

### SNP genotyping

Genomic DNA was extracted from 200 μL of each whole blood sample using a QIAamp DNA Blood Mini Kit (QIAGEN, Hilden, Germany) according to the manufacturer’s instructions. The DNA samples were genotyped for *ABCC2* C-24T (rs717620), *ABCC2* G1429A (rs2273697), and *ABCC4* T4976C (rs1059751) using TaqMan real-time PCR probes (Applied Biosystems, C___2814642_10C, C__22272980_20, and C___7461507_30, respectively). Deviations from Hardy-Weinberg equilibrium of the alleles and differences in the allele frequencies between cases and controls were evaluated by chi-square or Fisher’s exact test.

## Results

### Thai HIV patients receiving TDF for at least one year

A total of 273 HIV-1-infected individuals who had been receiving TDF for at least one year were enrolled in this study at the Bamrasnaradura Infectious Diseases Institute, Nonthaburi, Thailand, from February to December 2014. Patient demographic data are shown in Table 1. The study population consisted of 133 males (48.7%) and 140 females (51.3%) and the median body weight was 58 kg with interquartile range (IQR) of 50–64 kg. Most of the patients contracted infection through sexual contact. Median duration after HIV diagnosis was 13.6 years with IQR of 11.9–16.7 years. Two hundred and fifty-four of the 273 HIV patients had initiated ART without TDF and subsequently had changed to a combination including TDF. Median duration of ART was 11.4 years with IQR of 9.8–12.3 years, and the median duration of TDF-containing regimen was 5.04 years with IQR of 3.9–6.7 years. Only 19 of 273 HIV patients had been receiving TDF for less than 2 years.

**Table 1:** Demographic data for patient population.

*Demographic data*	n = *273*	
Median age (IQR) years	44	(39–48)
Male (%)	133	(48.7)
Median body weight (IQR) kg	58	(50–64)
Median duration after HIV diagnosis (IQR) years (n = 232)	13.6	(11.9–16.7)
Median Baseline CD4 (IQR) cells/μL (n = 186)	129	(30.5–212.5)
Median duration of therapy (IQR) years (n = 268)	11.4	(9.8–12.3)
Median CD4 at initiation of TDF regimen (IQR) cells/μL (n = 267)	421	(287.5–596.5)
Median duration of TDF regimen (IQR) years	5.04	(3.9–6.7)

TDF: Tenofovir disoproxil fumarate

IQR: Interquartile range

We first calculated eGFR to evaluate renal function, since prolonged exposure to TDF is also known to cause a more severe reduction of eGFR than ART regimens without TDF [6, 22, 25]. As expected, the eGFR values of the 273 patients were moderately and inversely correlated with patient age (r = -0.498). However, 222 of 273 patients exhibited eGFRs with normal range (>90 mL/min), 47 showed slight reduction (60–89 mL/min), and only four patients showed moderate reduction (50–59 mL/min). These results indicated that 98.5% of patients maintained eGFR despite extended exposure to TDF.

### Association of beta2-microglobulinuria with low body weight

The laboratory data of all patients are shown in Table 2; four known KTD parameters stratified for abnormal and normal groups are shown Table 3. Among the 273 patients, 54 (19.8%) showed beta2-microglobulinuria (median 2636.4 μg/g Cr, IQR 1519.1–13197.2 μg/g Cr). Thirty-three (12.1%) patients showed non-diabetic glucosuria (median 16.2 mg/dL, IQR 14.3–62.7 mg/dL). The FeP was increased in 13 (4.8%) patients (median 23.4%, IQR 20.4–28.6%) and Tmp/eGFR was decreased in 141 (51.6%) patients (median 2.4 mg/dL, IQR 2.2–2.6 mg/dL). Various numbers of patients showed abnormality among these four parameters of KTD.

**Table 2:** Clinical data for patient population (N = 273).

Tests	Normal range	Median	(IQR)
Urine protein (mg/dL)	1–15	8	(5–15.4)
Urine glucose (mg/dL)	< 12	5.79	(3–8.01)
Creatinine (mg/dL)	0.51–0.95 (F), 0.67–1.17 (M)	0.77	(0.65–0.92)
Phosphate (mg/dL)	2.7–4.5	3.1	(2.7–3.4)
Urine phosphate (mg/dL)	40–140	34.9	(20.3–55.1)
Urine creatinine (Cr) (mg/dL)	28–217	90.9	(56.47–148.12)
FeP (%)	< 20	9.6	(6.9–13.1)
Tmp/eGFR (mg/dL)	2.8–4.4	2.7	(2.4–3.1)
B2MG (mg/L)	< 0.15	0.19	(0.08–0.51)
B2MG/Cr (μg/g Cr)	<1000*	188.2	(107.7–642.9)

IQR: Interquartile range

F: Female

M: Male

FeP: Fractional excretion of phosphate

Tmp/eGFR: Tubular maximal transport of phosphate/estimated glomerular filtration rate

B2MG: Urine beta2-microglobulin

*: Nishijima et al. 2012.[22]

**Table 3:** Clinical data stratified for abnormal and normal group.

	Abnormal			Normal		
Tests	N	Median	(IQR)	N	Median	(IQR)
Urine glucose (mg/dL)	33	16.2	(14.3–62.7)	240	5.0	(3.0–7.0)
FeP (%)	13	23.4	(20.4–28.6)	260	9.2	(6.8–12.8)
Tmp/eGFR (mg/dL)	141	2.4	(2.2–2.6)	132	3.2	(2.9–3.4)
B2MG/Cr (μg/g Cr)	54	2636.4	(1519.1–13197.2)	219	145.3	(93.1–255.1)

IQR: Interquartile range

FeP: Fractional excretion of phosphate

Tmp/eGFR: Tubular maximal transport of phosphate/estimated glomerular filtration rate

B2MG/Cr: Urine beta2-microglobulin/creatinine

Since low body weight has been reported as a risk factor for TDF-induced KTD [21, 22], we compared body weights between patients with normal and abnormal values (Table 4). Among the four parameters, only beta2-microglobulinuria was associated with lower body weight (median 54.0 kg with IQR of 49–62 kg in the 54-patient beta2-microglobulinuria group vs. median 58.2 kg with IQR of 50–65 kg in the 219-patient normal group, p = 0.021, Mann-Whitney U-test). The median duration of TDF-containing ART in the 54-patient beta2-microglobulinuria group (5.4 years with IQR of 3.9–6.9 years) was statistically indistinguishable from that in the normal group (5.0 years with IQR of 3.9–6.6 years).

**Table 4:** Body weight and clinical data stratified for abnormal and normal group.

	Abnormal			Normal			
		Body weight (kg)			Body weight (kg)		
Tests	N	Median	(IQR)	N	Median	(IQR)	P value
Urine glucose (mg/dL)	33	57	(52–62)	240	58	(50–64)	0.421
FeP (%)	13	53	(44–62)	260	58	(50–64)	0.072
Tmp/eGFR (mg/dL)	141	57.8	(50–63)	132	58	(51–65)	0.774
B2MG/Cr (μg/g Cr)	54	54	(49–62)	219	58.2	(50–65)	0.021

IQR: Interquartile range

FeP: Fractional excretion of phosphate

Tmp/eGFR: Tubular maximal transport of phosphate/estimated glomerular filtration rate

B2MG/Cr: Urine beta2-microglobulin/creatinine

### Association of *ABCC4* 4976C allele with TDF-induced beta2-microglobulinuria

Since the beta2-microglobulinuria was the only KTD parameter that showed a significant association with low body weight, we stratified genotyping results of *ABCC2* C-24T (rs717620), *ABCC2* G1429A (rs2273697), and *ABCC4* T4976C (rs1059751) according to the presence or absence of beta2-microglobulinuria (Table 5). All polymorphisms were in Hardy-Weinberg equilibrium with a cutoff P value of 0.05. The allele frequency of *ABCC4* 4976C among the 54 patients with beta2-microglobulinuria was 0.602, while it was 0.475 among 219 patients without beta2-microglobulinuria (p = 0.018). The frequency of the CC genotype was nominally higher in the 54 patients with beta2-microglobulinuria (0.370) than in the 219 patients without beta2-microglobulinuria (0.242), but this difference did not reach statistical significance (p = 0.056). Among the 54 patients with beta2-microglobulinuria, 35 were CC homozygotes, while the remaining 19 were CT heterozygotes or TT homozygotes. There was a gradual increase in the number of patients with beta2-microglobulinuria when comparing TT homozygotes, CT heterozygotes, and CC homozygotes within the studied population, with proportions of 0.123, 0.197, and 0.273, respectively. These results indicated that the C allele at position 4976 of the *ABCC4* gene was associated with beta2-microglobulinuria in the studied population of patients. In contrast, the frequencies of *ABCC2* -24C and *ABCC2* 1429A alleles, which were previously shown to be overrepresented in HIV patients with KTD receiving TDF (18, 20), were not overrepresented in our beta2-microglobulinuria group (Table 5). Instead, the *ABCC2* 1429A allele showed a tendency towards higher frequency in the normal control group. However, this trend did not reach statistical significance (p = 0.127) although the allele frequency of *ABCC2* 1429A in the control group (9.8%) was more than twice that in the beta2-microglobulinuria group (4.6%).

**Table 5:** SNP genotype among 54 patients with beta2-microglobulinuria (case) and others (control).

*ABCC2* C-24T (rs717620)				*ABCC2* G1429A (rs2273697)				*ABCC4* T4976C (rs1059751)			
	Case	Control	P^1^		Case	Control	P^2^		Case	Control	P^1^
CC	35	145		GG	49	178		TT	9	64	
CT	16	63		GA	5	39		TC	25	102	
TT	3	11		AA	0	2		CC	20	53	
Total	54	219		Total	54	219		Total	54	219	
C allele	0.796	0.806	0.821	A allele	0.046	0.098	0.127	C allele	0.602	0.475	0.018
CC genotype	0.648	0.662	0.846	AA genotype	0	0.009	1.000	CC genotype	0.37	0.242	0.056

^1^: Chi^2^ test

^2^: Fisher’s exact test

When we stratified genotyping results according to the presence or absence of abnormality in urine glucose, FeP%, or Tmp/eGFR, we found that none of these parameters were significantly associated with the C allele at position 4976 of the *ABCC4* gene (Tables A, B, and C in S1 File). Clinically relevant KTD is usually diagnosed when at least two of those parameters show abnormal values. Therefore, we stratified genotyping results according to the presence or absence of beta2-microglobulinuria and at least one more abnormality in urine glucose, FeP%, or Tmp/eGFR (Table D in S1 File). This stratification yielded the same trend as that seen for beta2-microglobulinuria alone, but the results did not reach statistical significance (p = 0.151).

## Discussion

We performed our analysis in a study population consisting of Thai HIV patients who had been receiving TDF for at least one year. Approximately 20% of these patients had beta2-microglobulinuria, while only 1.5% showed decreased eGFR. Beta2-microglobulinuria was associated with lower patient body weight. In addition, we found that the *ABCC4* 4976C allele was associated with beta2-microglobulinuria in this population. This polymorphism may help to identify patients at greater risk of developing TDF-associated KTD, especially among patients who have been stably receiving TDF.

Higher plasma concentration of tenofovir [26, 27] and lower patient body weight [22, 28] were reported to be associated with TDF-induced KTD. A recent report by Calcagno et al. showed that the CC genotype of the *ABCC4* 4976 was associated with markedly decreased urinary exclusion of tenofovir, although plasma concentration of tenofovir did not differ between different genotypes at *ABCC4* 4976 [29]. It is possible that the *ABCC4* 4976C allele is associated with impaired exclusion of tenofovir into urine, resulting in more concentrated tenofovir in tubular cells, which might lead to KTD in TDF-receiving patients. However, further studies will be required to elucidate the precise mechanism underlying the fact that the *ABCC4* 4976C allele was associated with beta2-microglobulinuria.

On the other hand, we failed to detect any association between the *ABCC2* -24C or 1429A alleles and beta2-microglobulinuria, even though several previous studies reported an association of these alleles with TDF-related KTD [20, 22]. This discrepancy may reflect the fact that our patients constituted a patient group that had been receiving TDF for a relatively long interval without decreases of eGFR. In contrast, KTD has been reported to occur in numerous cases after 0.6 to 2.5 years of TDF exposure [20, 22, 25]. It should be noted here that it is a common practice in the hospital to stop TDF administration if patients show renal dysfunction after initiation on TDF-containing drugs. In addition, most of our patients (255 out of 273) were treatment-experienced individuals for whom TDF was not the first anti-retroviral drug prior to being placed on a TDF-containing regime and had subsequently been stably receiving TDF. Therefore, our study population was composed of patients who showed a certain degree of tolerance to TDF, which might explain the discrepancy between previous studies and ours.

The apparent disadvantage of our study on patients with at least 1 year of TDF use is that patients with significant toxicity might already have dropped out before inclusion. The previous retrospective study in the Bamrasnaradura Infectious Diseases Institute showed that among 3,154 HIV patients initiated on TDF during the period of January 1^st^ 2007 to October 31^st^ 2010, 112 patients (3.55%) stopped TDF due to nephrotoxicity, including both KTD and glomerular dysfunction [30]. These data suggest that our study would have failed to enroll a rather small patient proportion. However, Lubomirov et al. reported that TDF-receiving HIV patients homozygous for *ABCC2* 1429A tended to stop TDF within 1 year, a shorter interval than that seen for patients with other genotypes of this SNP [31]. This difference might explain why our study detected this allele at lower frequencies in patients with beta2-microglobulinuria.

We observed an association between the *ABCC4* 4976C allele and beta2-microglobulinuria. Beta2-microglobulinuria is widely used to detect KTD. In the present study, 12 out of 13 patients with increased FeP had beta2-microglobulinuria, while 16 out of 33 patients with glucosuria had beta2-microglobulinuria. These results suggested that KTD is multifactorial and that the mechanism of glucosuria may be different from that of beta2-microglobulinuria. These results also might explain why the association of *ABCC4* 4976C with clinically relevant KTD became weaker than that with beta2-microglobulinuria (Table D in S1 File). In addition, we found four patients with eGFR <60 mL/min. Among these four individuals, two had glucosuria, and two showed increased FeP%, but all four showed decreased Tmp/eGFR and beta2-microglobulinuria. Although our data showed that beta2-microglobulinuria is a sensitive candidate marker of KTD, it still remains unclear which parameter will best permit early detection of treatment-limiting KTD. Medical personnel will need to monitor kidney function periodically and continuously during treatment, including that with TDF.

## Supporting Information

S1 File Table A in S1 File. SNP genotype among 33 patients with high urine glucose (case) and others (control). Table B in S1 File. SNP genotype among 13 patients with high FeP (case) and others (control). Table C in S1 File. SNP genotype among 141 patients with low Tmp/eGFR (case) and others (control). Table D in S1 File. SNP genotype among 41 cases with beta-2 microgloblinurira and at least one more abnormality (case) and others (control) (DOCX)
