# Supplementary material for: A Single-Nucleotide Polymorphism in ABCC4 Is Associated with Tenofovir-Related Beta2-Microglobulinuria in Thai Patients with HIV-1 Infection
Source: PLoS One. 2016 Jan 25;11(1):e0147724. doi: 10.1371/journal.pone.0147724 (PMC4726597; doi:10.1371/journal.pone.0147724)
Supplement: S1 File — (DOCX) [file pone.0147724.s001.docx]

Table A. SNP genotype among 33 patients with high urine glucose (case) and others (control)

|  |  |  |  |  |  |  |  |  |  |  |  |  |  |
| --- | --- | --- | --- | --- | --- | --- | --- | --- | --- | --- | --- | --- | --- |
| ABCC2 C-24T (rs717620) | | | |  | ABCC2 G1429A (rs2273697) | | | |  | ABCC4 T4976C (rs1059751) | | | |
|  | Case | Control | P^1^ |  |  | Case | Control | P^2^ |  |  | Case | Control | P^1^ |
| CC | 16 | 164 |  |  | GG | 31 | 196 |  |  | TT | 9 | 64 |  |
| CT | 16 | 63 |  |  | GA | 2 | 42 |  |  | TC | 16 | 111 |  |
| TT | 1 | 13 |  |  | AA | 0 | 2 |  |  | CC | 8 | 65 |  |
| Total | 33 | 240 |  |  | Total | 33 | 240 |  |  | Total | 33 | 240 |  |
| C allele | 0.727 | 0.815 | 0.094 |  | A allele | 0.030 | 0.096 | 0.102 |  | C allele | 0.485 | 0.502 | 0.793 |
| CC genotype | 0.485 | 0.683 | 0.024 |  | AA genotype | 0 | 0.008 | 1.000 |  | CC genotype | 0.242 | 0.271 | 0.730 |

^1^:Chi^2^ test

^2^:Fisher’s exact test

Table B. SNP genotype among 13 patients with high FeP (case) and others (control)

|  |  |  |  |  |  |  |  |  |  |  |  |  |  |
| --- | --- | --- | --- | --- | --- | --- | --- | --- | --- | --- | --- | --- | --- |
| ABCC2 C-24T (rs717620) | | | |  | ABCC2 G1429A (rs2273697) | | | |  | ABCC4 T4976C (rs1059751) | | | |
|  | Case | Control | P^1^ |  |  | Case | Control | P^1^ |  |  | Case | Control | P^1^ |
| CC | 9 | 171 |  |  | GG | 11 | 216 |  |  | TT | 2 | 71 |  |
| CT | 3 | 76 |  |  | GA | 1 | 43 |  |  | TC | 7 | 120 |  |
| TT | 1 | 13 |  |  | AA | 1 | 1 |  |  | CC | 4 | 69 |  |
| Total | 13 | 260 |  |  | Total | 13 | 260 |  |  | Total | 13 | 260 |  |
| C allele | 0.808 | 0.804 | 1.000 |  | A allele | 0.115 | 0.087 | 0.491 |  | C allele | 0.577 | 0.496 | 0.547 |
| CC genotype | 0.692 | 0.657 | 1.000 |  | AA genotype | 0.077 | 0.004 | 0.093 |  | CC genotype | 0.308 | 0.265 | 0.752 |

FeP: fraction excretion of phosphate

^1^: Fisher’s exact test

Table C. SNP genotype among 141 patients with low Tmp/eGFR (case) and others (control)

|  |  |  |  |  |  |  |  |  |  |  |  |  |  |
| --- | --- | --- | --- | --- | --- | --- | --- | --- | --- | --- | --- | --- | --- |
| ABCC2 C-24T (rs717620) | | | |  | ABCC2 G1429A (rs2273697) | | | |  | ABCC4 T4976C (rs1059751) | | | |
|  | Case | Control | P^1^ |  |  | Case | Control | P^2^ |  |  | Case | Control | P^1^ |
| CC | 88 | 92 |  |  | GG | 117 | 110 |  |  | TT | 38 | 35 |  |
| CT | 45 | 34 |  |  | GA | 23 | 21 |  |  | TC | 68 | 59 |  |
| TT | 8 | 6 |  |  | AA | 1 | 1 |  |  | CC | 35 | 38 |  |
| Total | 141 | 132 |  |  | Total | 141 | 132 |  |  | Total | 141 | 132 |  |
| C allele | 0.784 | 0.826 | 0.216 |  | A allele | 0.089 | 0.087 | 1.000 |  | C allele | 0.489 | 0.511 | 0.607 |
| CC genotype | 0.624 | 0.697 | 0.204 |  | AA genotype | 0.007 | 0.008 | 1.000 |  | CC genotype | 0.248 | 0.288 | 0.460 |

Tmp/eGFR: tubular maximal transport of phosphate/estimated glomerular filtration rate

^1^: Chi^2^ test

^2^: Fisher’s exact test

Table D. SNP genotype among 41 cases with beta-2 microgloblinurira and at least one more abnormality (case) and others (control)

|  |  |  |  |  |  |  |  |  |  |  |  |  |  |
| --- | --- | --- | --- | --- | --- | --- | --- | --- | --- | --- | --- | --- | --- |
| ABCC2 C-24T (rs717620) | | | |  | ABCC2 G1429A (rs2273697) | | | |  | ABCC4 T4976C (rs1059751) | | | |
|  | Case | Control | P^1^ |  |  | Case | Control | P^2^ |  |  | Case | Control | P^1^ |
| CC | 25 | 155 |  |  | GG | 37 | 190 |  |  | TT | 8 | 65 |  |
| CT | 13 | 66 |  |  | GA | 4 | 40 |  |  | TC | 19 | 108 |  |
| TT | 3 | 11 |  |  | AA | 0 | 2 |  |  | CC | 14 | 59 |  |
| Total | 41 | 232 |  |  | Total | 41 | 232 |  |  | Total | 41 | 232 |  |
| C allele | 0.769 | 0.810 | 0.377 |  | A allele | 0.049 | 0.095 | 0.209 |  | C allele | 0.573 | 0.487 | 0.151 |
| CC genotype | 0.610 | 0.668 | 0.467 |  | AA genotype | 0 | 0.009 | 1.000 |  | CC genotype | 0.341 | 0.254 | 0.245 |

^1^: Chi^2^ test

^2^: Fisher’s exact test
